# Multi-Source Data Fusion Improves Time-Series Phenotype Accuracy in Maize under a Field High-Throughput Phenotyping Platform

**DOI:** 10.34133/plantphenomics.0043

**Published:** 2023-04-20

**Authors:** Yinglun Li, Weiliang Wen, Jiangchuan Fan, Wenbo Gou, Shenghao Gu, Xianju Lu, Zetao Yu, Xiaodong Wang, Xinyu Guo

**Affiliations:** ^1^ Information Technology Research Center, Beijing Academy of Agriculture and Forestry Sciences, Beijing 100097, China.; ^2^ Beijing Key Lab of Digital Plant, National Engineering Research Center for Information Technology in Agriculture, Beijing 100097, China.

## Abstract

The field phenotyping platforms that can obtain high-throughput and time-series phenotypes of plant populations at the 3-dimensional level are crucial for plant breeding and management. However, it is difficult to align the point cloud data and extract accurate phenotypic traits of plant populations. In this study, high-throughput, time-series raw data of field maize populations were collected using a field rail-based phenotyping platform with light detection and ranging (LiDAR) and an RGB (red, green, and blue) camera. The orthorectified images and LiDAR point clouds were aligned via the direct linear transformation algorithm. On this basis, time-series point clouds were further registered by the time-series image guidance. The cloth simulation filter algorithm was then used to remove the ground points. Individual plants and plant organs were segmented from maize population by fast displacement and region growth algorithms. The plant heights of 13 maize cultivars obtained using the multi-source fusion data were highly correlated with the manual measurements (*R*^2^ = 0.98), and the accuracy was higher than only using one source point cloud data (*R*^2^ = 0.93). It demonstrates that multi-source data fusion can effectively improve the accuracy of time series phenotype extraction, and rail-based field phenotyping platforms can be a practical tool for plant growth dynamic observation of phenotypes in individual plant and organ scales.

## Introduction

The increasing global population and decreasing area of arable land per capita have affected world food security [1,2]. Therefore, new, efficient breeding methods should be developed to increase crop yields [3]. Molecular breeding with fast, accurate, and stable features has been improved due to the development of next-generation sequencing technology [4]. However, genomic studies cannot satisfactorily and genetically improve complex traits controlled by biotic and abiotic factors because of the lack of high-throughput and accurate phenotypic data for the discovery, identification, and selection of genetic loci [4,5].

Traditional crop phenotyping studies are time-consuming and destructive, and cannot be monitored on a regional scale. However, researchers can achieve automated, high-throughput access to phenotypic information through multiple data sources, such as crop images [6–9], point clouds [10–12], and spectra collected in the field because of the continuous development of sensor technology and artificial intelligence in agricultural applications [1,2 ,5]. However, most existing phenotypic studies focus on monitoring structural traits at key growth stages, which lack time-series information and do not reflect the physiological mechanisms of plant growth and development. Plants are dynamically changing organisms that increase physiological and structural complexity as they grow [13]. Plant phenotypic traits are associated with temporal and topological changes during development and constantly change throughout their life cycle. The structure and evolution of plants are of agronomic importance since they determine the efficiency of resource use and plant performance [14,15]. Therefore, it is crucial to analyze the dynamics of phenotypic traits during plant growth and development to understand the genetic control of traits and their response to the environment.

The high-throughput phenotyping (HTP) platform enables the regular collection of multiple sources of heterogeneous raw data on many genotyped plants in the field environment [16]. This platform mainly uses RGB cameras and LiDAR as sensors [17–19]. The RGB camera captures high-resolution images that can be used to extract various traits, such as canopy structure, the number and morphology of specific organs for growth stage identification [20,21], organ density analysis [22], and the estimation of the Green Area Index during growth [23]. However, it is difficult to obtain accurate estimation accuracy using only 2-dimensional (2D) images since plants exist in 3 dimensions. However, RGB cameras are sensitive to light conditions due to the limitations of the large field environment. In contrast, LiDAR is not affected by light conditions. As a result, the high-density point cloud information acquired by LiDAR can provide a detailed description of the 3-dimensional (3D) structure of the target canopy [18,24]. LiDAR-based phenological studies mainly depend on the representativeness of the features extracted from the 3D point cloud. Canopy height and profile distribution information are the essential point cloud features. As a result, studies have used the 2 parameters to improve the accuracy of canopy biomass estimation [23,25 ,26]. Nevertheless, previous research results among different growth stages cannot be generalized. Besides, the method is less robust when used in other cultivars under different growing conditions and thus requires additional improvements. The focus of phenotype extraction using time-series point cloud data acquired through linear LiDAR is based on point cloud alignment and segmentation [27–31]. Alignment is crucial for normalizing point cloud data from different growth and development stages to the same spatial coordinate system for phenotypic extraction. Segmentation is vital for obtaining phenotypic parameters using 3D point clouds [32,33]. However, the accuracy of adaptive alignment using only time-series point cloud data is often insufficient for phenotype extraction. Therefore, the advantages of RGB cameras and LiDAR can be combined to monitor crop phenotypes and improve the accuracy of the corresponding phenotypic parameters [34,35]. The HTP platform can be used to simultaneously acquire RGB images of the field and 3D LiDAR data. The multi-temporal point cloud data can be aligned using the feature points in the 2D orthorectified image as critical points. The 2D images can be used to link time-series information from 3D point clouds. Furthermore, automated segmentation algorithms can be used to resolve temporal changes in plant populations, providing a powerful method for monitoring dynamic changes in crop phenotypic parameters.

In summary, phenotype algorithm development is limited by the difficulty in mining multi-source time-series phenotype data with high information dimensions. In this study, a highly time-series accurate phenotypic parameter extraction method of maize was used for data collection from field phenotyping platforms. The 3D point cloud data were aligned using orthorectified images of the trial field simultaneously acquired using the field phenotyping platform. The 2D images and 3D point cloud data were used to achieve dynamic phenotype monitoring in high time series. The method had good scalability and could provide a practical technical tool for high-throughput, high time-series phenotype extraction.

## Materials and Methods

### Experimental design and data acquisition

**Fig. 1.**
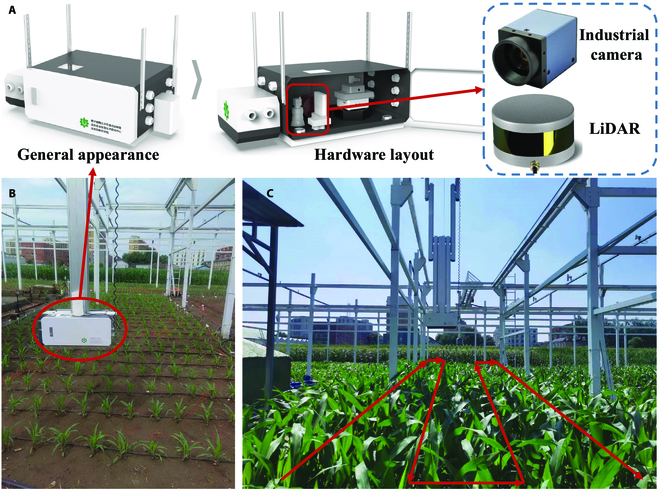
Study area and data collection using a rail-based phenotyping platform. (A) Stabilizing of the imaging chamber with its mounted industrial camera and Velodyne 16 LIDAR sensor. (B) The rail-based phenotyping platform. (C) Path planning during the operation of the phenotyping platform.

The study area was located at the experimental field in Beijing Academy of Agriculture and Forestry (39°56′N, 116°16′E), Beijing, China. Data collection in the field was conducted using a rail-based phenotyping platform [36,37]. The platform was 15 m long, 4 m wide, and 5 m high, covering an area of about 60 m^2^. Thirteen cultivars of maize (including AD268, MC670, JNK2010, JNK728, NK815, JKQC516, SK567, Y968, MC141, ZD958, XY335, JK968, and M751) were planted (June 25) within the coverage of the rail-based phenotypic platform, with one protecting row on each side. Each variety was planted in one row (3 m long, the seeds were planted 60 cm apart, and maize plants in each row were 30 cm apart). Drip irrigation was conducted to ensure adequate water and fertilizer. The platform's phenotype bin had an RGB camera and a LiDAR with an S-shaped trajectory over the field during the data collection process. The platform was used to simultaneously acquire the top view images and 3D point cloud data of maize populations in the field for about 20 min. Data collection was conducted daily for 1 week after sowing. The data from D10 (10 days after emergence) to D65 (65 days after emergence) (scanned at 3 time points; 8:00, 12:00, and 16:00) were used. The dataset contained maize development from V3 to R6 [38]. The specific data collection process is shown in Fig. 1.

A Hikvision MV-CA060-10GC color camera (1/1.8" CMOS Gigabit Ethernet industrial camera with 6 megapixels; resolution, 3,072 × 2,048; lens, 4 m from the ground) was used to capture the top-view images. The platform collected 270 images per trip. Velodyne 16 LiDAR (Velodyne, California, USA) was used to obtain point cloud data, and it could achieve data output of up to 300,000 points per second with a measurement range of up to 100 m. The LiDAR lens was 4 m from the ground. More detailed parameters for sensors are shown in Table.

**Table.** The performance parameters for sensors.

	Camera	LiDAR
Name	Hikvision MV-CA060-10GC	Velodyne 16
Shooting mode	Automatic	Automatic
Spatial resolution	0.4–0.5 mm/pix	Laser wavelength: 905 nm
Operating temperature (°C)	−30–70	−10–60
Interface type	GigE	GigE
External size	29 mm × 29 mm × 42 mm	103 mm × 72 mm
Data resolution	3,072 × 2,048	300,000 points/s

### Data pre-processing and time-series point cloud alignment

**Fig. 2.**
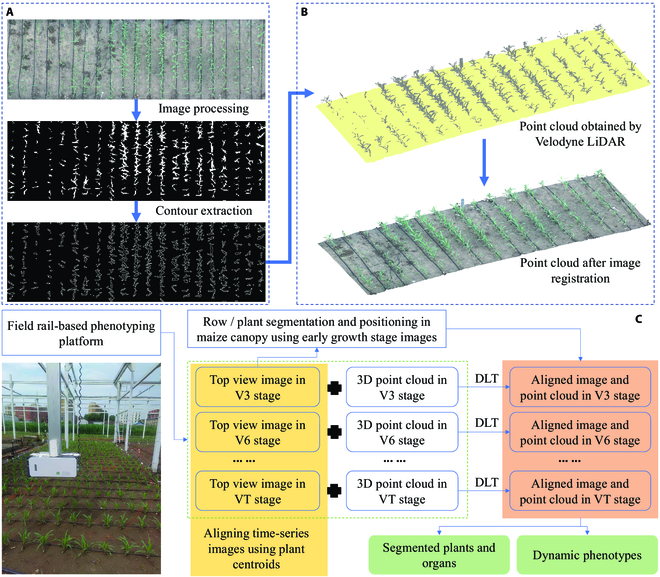
Alignment of point clouds using image guidance. (A) Image processing process (orthorectified images of maize populations in the field after processing with OpenDroneMap software, binarized images, and plant profile extraction results). (B) Point cloud obtained from Velodyne (top half) and an example of the image-aligned point cloud (bottom half). (Examples of seedling images and time-series point cloud are aligned according to the process.) (C) Overview of time-series data alignment using time-series image alignment and data fusion.

The reconstructed maize population point clouds were aligned using the field maize population orthorectified images. Open-source software OpenDroneMap (https://github.com/OpenDroneMap) was used to stitch the orthorectified images of maize populations (Fig. 2A). The orthorectified images were generated using OpenDroneMap. The images were then segmented and extracted using the PlantU-net model [39]. The 3D point cloud data were solved based on parameters such as trajectory of sensors, time, and speed. The initial alignment of each scan was rapidly completed using matrix transformation of the standard WGS84 coordinate system. Reconstruction was then performed to obtain high-density point cloud data of the maize population in the field (Fig. 2B). Figure 2C shows an overview of time-series data alignment using time-series images alignment and data fusion.

The image in Fig. 2A only presents specific areas. The HTP platform arranged calibration objects (e.g., spheres or squares) at the start position of each run. The calibration objects could be used to align images from different growth stages. In addition, the outline of the maize plant after image segmentation also provides a basis for alignment. The high frequency of time-series data acquisition ensures that sufficient feature points can be found between adjacent data for image alignment.

The point clouds of maize populations from different periods were resolved using coordinate deviations. The orthorectified images were aligned (orientation correction and scaling) with the point clouds obtained from the LiDAR scans via the direct linear transformation (DLT) [40] method. An alignment was performed by establishing a direct linear relationship between the planar coordinates of critical points in the image and the spatial coordinates of issues in the corresponding 3D point cloud. The DLT is a simplified linear model of the standard line equation with the following specific expressions:

$$x+\frac{l_{1}X+l_{2}Y+l_{3}Z+l_{4}}{l_{9}X+l_{10}Y+l_{11}Z+1}=0$$ (1)

$$y+\frac{l_{5}X+l_{6}Y+l_{7}Z+l_{8}}{l_{9}X+l_{10}Y+l_{11}Z+1}=0$$ (2)

where (*x*, *y*) represent the 2D coordinate parameters of the feature points in the image; (*X*, *Y*, *Z*) represent the spatial coordinate parameters of the feature points in the 3D point cloud at known locations; and *l*_1_ to *l*_11_ represent the 11 sets of unknown parameters needed to calibrate the model.

The orthophoto image of the top view image of the maize group in the field may be distorted as a non-linear factor during the stitching process. Therefore, the (*x*, *y*) coordinate parameters in the image can be expressed as:

$$x=\mathrm{fx}\left(l_{1},l_{2},l_{3},l_{4},l_{5},l_{6},l_{7},l_{8},l_{9},l_{10},l_{11},X,Y,Z\right)$$ (3)

$$y=\mathrm{fy}\left(l_{1},l_{2},l_{3},l_{4},l_{5},l_{6},l_{7},l_{8},l_{9},l_{10},l_{11},X,Y,Z\right)$$ (4)

The above expression can be linearly expanded if there are *n* critical points, thus obtaining the following equation:

x1y1x2y2...xnyn+X10X20...Xn0Y10Y20...Yn0Z10Z20...Zn01010...100X10X2...0Xn0Y10Y2...0Yn0Z10Z2...0Zn0101...01x1X1y1X1x2X2y2X2...xnXnynXnx1Y1y1Y1x2Y2y2Y2...xnYnynYnx1Z1y1Z1x2Z2y2Z2...xnZnynZnl1l2l3l4l5l6l7l8l9l10l11=0 (5)

The DLT model has 11 unknown parameters, indicating that 6 sets of feature points are required for alignment parameter estimation. The corresponding error equations and normal equations were as follows:

V=BL−W (6)

$$L=\left(\text{BTPB}\right)-1\text{BTPW}$$ (7)

Feature point extraction is crucial in the alignment process. Herein, the orthorectified images were binarized as described by Li et al. [41]. The contours of the maize plants and the centroids were used as crucial feature points in the orthorectified images (Fig. 2A). The point cloud was projected as a flat image using parallel projection. The matching features were identified following the image processing steps. The feature points in the orthomosaic and point cloud projection images were then grouped based on a rough distribution pattern, with 2 points in each group (the 2 closest feature points, one from the ortho-image and the other from the point cloud projection image). Alignment of image and point cloud data was performed based on the correspondence of the matched feature points. The feature points were derived by matching the contours in orthorectified images and the *z*-axis projection of the point cloud. The number of feature points after matching should be greater than 11 for the calculation of DLT.

### Ground point cloud removal

The KD-tree [42] method was used to remove noise points from the data to obtain a high-quality, aligned point cloud. The ground point cloud was removed using the cloth simulation filtering (CSF) algorithm [41,43] to accurately extract phenotypic traits in the population. This is difficult to achieve because of the uneven ground in the field and the presence of imaging factors, such as drip irrigation strips. The algorithm inverts the point cloud, assuming that a piece of fabric is overlaid on the flipped point cloud. The final form after fabric coverage represents the ground point cloud in the maize population point cloud in the field. This formula is summarized as follows:

$$m\frac{\partial A\left(t\right)}{\partial t^{2}}=F_{\mathrm{ext}}\left(A,t\right)+F_{\mathrm{int}}\left(A,t\right)$$ (8)

*A* represents the position of the particle in the cover at moment *t*; *F_ext_*(*A*, *t*) represents the external driver; *F_int_*(*A*, *t*) represents the internal driver.

The algorithm cover was imaged by both *F_ext_*(*A*, *t*) and *F_int_*(*A*, *t*) factors. The points were projected to the same level as the particles in the mulch by flipping the aligned point cloud. The displacement of the particles by the gravity image and internal factor image was also calculated. The effect of ground point cloud removal was achieved until the threshold of the previous folding was met or when the number of iterations reached zero.

### Point cloud segmentation and phenotype parameter extraction

The maize population point cloud was segmented using a Quickshift algorithm [44,45] based on density information adjustment and an area growth algorithm [46]. The 3D point cloud data were projected onto the ground plane before clustering, and then the vertical structure was separated. The position of the maize stems was determined using the 2D projection of the point cloud density. The stems and leaves in the maize point cloud were further segmented. The 2D projection was used to define the clustering information for the stems and leaves. The clustering of leaves in 3D space (clustering leaves into multiple subclasses along the stem) was conducted to solve the problem of overlapping and obscuring leaves in the 3D point cloud. Specifically, clustering was first performed using a fast displacement algorithm to initialize the densest region of nearby points in 2D density space. The remaining points were then clustered in a 3D area to ensure that only issues that were close in 3D space or extremely dense regions (possibly stems) in the 2D projection were in the same cluster. The initialization process does not require a distance parameter, indicating that the algorithm was entirely independent of the size of the point cloud and was very robust. Phenotypic parameters, such as plant height, leaf inclination, and azimuth, were extracted based on the segmentation results as described by Li et al. [12].

### Assessment indicators

The plant heights extracted from the point clouds were compared with those obtained via manual measurements. The manual measurements represented accurate results. The height of all plants was measured every day. The heights of the same varieties were averaged and used as a representative value for the plant height. The comparison results were assessed using the correlation coefficient (*R*^2^), the mean square error (MSE), the root mean square error (RMSE), and the mean absolute error (MAE) as follows:

$$R^{2}=1-\frac{\sum_{l=1}^{m}{\left(v_{l}-v_{l}^{\prime }\right)}^{2}}{\sum_{l=1}^{m}{\left(v_{l}-{\bar{v}}_{l}\right)}^{2}}$$ (9)

$$\mathrm{MSE}=\frac{1}{m}\sum_{l=1}^{m}{\left(v_{l}-{\bar{v}}_{l}\right)}^{2}$$ (10)

$$\text{RMSE}=\sqrt{\frac{1}{m}\sum_{l=1}^{m}{\left(v_{l}-v_{l}^{\prime }\right)}^{2}}$$ (11)

$$\mathrm{MAE}=\frac{1}{m}\sum_{l=1}^{m}\left|v_{l}-{\bar{v}}_{l}\right|$$ (12)

where *m* represents the number of comparison objects, *v_l_* represents the value of the manual measurement results, $v_{l}^{\prime }$ represents the value of the phenotypic parameter extracted from the automatic segmentation result using the algorithm, and ${\bar{v}}_{l}$ represents the mean value of the manual measurement result.

## Results

### Multi-source data fusion

The experiments were conducted using a Windows 10, 64-bit workstation configured with an Intel(R) Xeno(R) Gold 6148 CPU @ 2.4 GHz 2.39 GHz × 2 processors with 256 GB RAM and an NVIDIA Quadro P6000 × 2. Two CPUs with 40 cores and 80 threads of computing power, combined with 2 NVIDIA Quadro P6000s with 48 GB of video memory, can reduce the time required for image and point cloud processing.

**Fig. 3.**
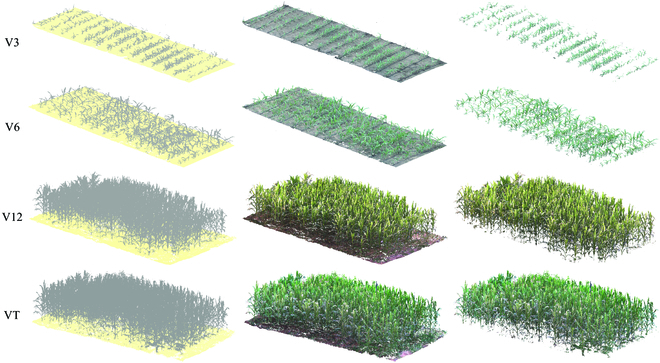
Visualization of point clouds before and after multi-source data fusion. The left and middle images represent the original point cloud and fused point cloud, respectively. The original point cloud contained only spatial coordinate information (the ground has been colored for presentation purposes). The ground point cloud removal from maize population point clouds at different growth stages is shown on the right.

The standard process is shown in Fig. 2. The time-series images were fused with the point cloud data. The visualization of the time-series point cloud after multi-source data fusion is shown in Fig. 3. The point cloud obtained via the linear LiDAR scan had detailed information, including color. Moreover, the point clouds acquired at different days were aligned to a uniform spatial coordinate system from a time-series perspective, which is crucial for parsing time-series data.

The results of the alignment and removal of ground point clouds from the maize population point clouds in the field at different growth stages are shown in Fig. 3 (right). The CSF algorithm effectively removed ground points from the maize population point cloud in the field. The algorithm only considered the downward tension of the coverings, thus automatically eliminating the ground point cloud without much parameter settings and with robustness across different growth stages.

### Point cloud segmentation of maize at different growth stages

**Fig. 4.**
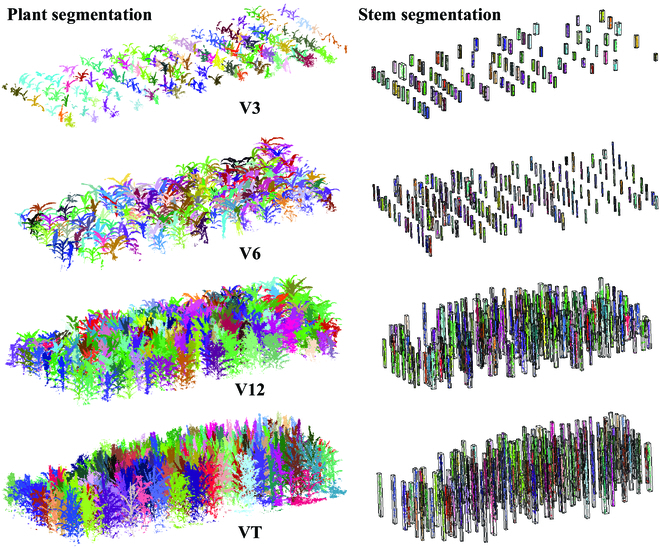
Visualization of point cloud segmentation.

The segmentation results of the point cloud of maize populations in several specific growth stages are shown in Fig. 4. The left and right images in the figure show the results of maize plant segmentation of each subplot and stem segmentation results, respectively. There were some blank areas in the stem segmentation results for the V3 period since some maize stems were not formed in the early stages. The stem segmentation results in Fig. 4 are presented based on the smallest enclosing box for each stem afterwards. As a result, some display enclosing packages look untidy with height differences. The segmentation results of the stems can provide a reliable basis for many studies, such as locating plant position and calculating field uniformity. The segmentation results of individual plants are crucial for extracting phenotypic parameters. The point cloud data from 10 to 65 days after seedling emergence were also analyzed and processed. Each set of point clouds was segmented into individual plants. Phenotypic parameters were extracted, and the temporal growth dynamics of maize were determined and analyzed.

### Effect of time-series point cloud alignment on phenotype extraction

**Fig. 5.**
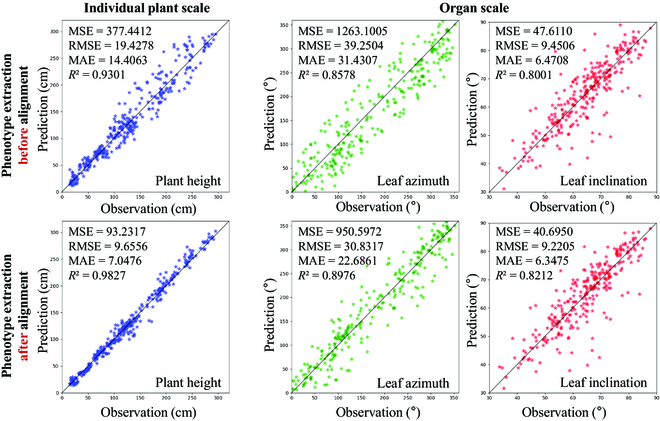
Comparison of plant height, leaf azimuth, and leaf inclination angle before and after point cloud data alignment.

Point cloud data alignment facilitates locating the individual plants in the population and thus improves the phenotype extraction accuracy. Figure 5 shows the comparison of plant height, leaf azimuth, and leaf inclination angle before and after point cloud data alignment. For plant height at the individual plant scale, point cloud segmentation determines the plant height estimation accuracy. If only a standalone point cloud was used for segmentation, nearby plant points might be segmented in other plants. In contrast, image and point cloud fusion data can be segmented using the growth position information of each individual plant in the canopy, which was derived from previous growth stage images and point clouds, thus improving the segmentation accuracy, and the *R*^2^ of plant height increased from 0.93 to 0.98. For organ scale phenotyping traits, since the organs near the stem of each plant can be identified, the accuracy of leaf azimuth and inclination angle was satisfactory for further applications. The *R*^2^ of leaf azimuth increased from 0.8578 to 0.8976 after data fusion, and the *R*^2^ of leaf inclination angle increased from 0.80 to 0.82. However, point clouds at the tip or far from the stems of many leaves were cut into other plants, leading the leaf length much shorter or longer than measurement. The present results demonstrate that multi-source data fusion could improve the accuracy in time-series phenotype estimation.

### Dynamics of structural traits for population and single maize

**Fig. 6.**
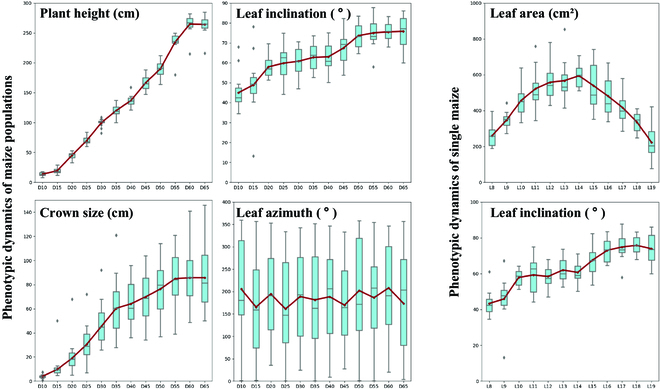
Phenotypic dynamics of populations and single maize. For maize populations, the *y*-axis represents the values of the corresponding phenotypic parameters, while the *x*-axis (D10 to D65) represents the number of days after seedling emergence. For single maize, the *y*-axis represents the values of the phenotypic parameters, while the *x*-axis (L8 to L19) represents the different leaf positions (the cob position leaves were labeled as L14).

The rail-based phenotyping platform collected high time-series data from maize populations for 55 consecutive days. The results of the analysis of the phenotypic dynamics of the 13 cultivars of maize plants every 5 days are shown in Fig. 6 (left). The leaf growth plane varied irregularly over the growth cycle of maize, making it difficult to find growth patterns. The other parameters increased with increasing time to emergence. Most of the parameters started to plateau 60 days after emergence, while the leaf crown size of maize entered a slow growth state 35 days after emergence.

The maize point clouds were also segmented during the D65 period to analyze phenotypic differences between leaf positions at the individual plant scale. For statistical analysis, the maize corm leaves were uniformly labeled as L14. The phenotypic parameters of the lower 6 leaves and the upper 5 leaves of the corm (12 leaves) were then determined. The phenotypic parameters are shown in Fig. 6 (right). Phenotypic parameters at different leaf positions of a single maize plant were similar to those of a parabola. Owing to the loss of point clouds at later stages, the lower leaves of some maize plants were extracted using time-series data. Only upper leaf information can be extracted using data from a single time point.

## Discussion

### Multi-source data fusion improves phenotype estimation accuracy

The results of the extraction of maize plant height for 13 cultivars in the test plots at different growth stages were highly correlated with the manual measurements (*R*^2^ = 0.98). This shows that the proposed method can be used to extract phenotypic parameters in maize populations in the field environment. This method can be used to conduct field phenotypic tests in a high-throughput manner. The multi-source data fusion process is crucial for time-series data analysis since it can effectively resolve the offset of point cloud data between periods, thus improving the accuracy of phenotype extraction. Some studies use depth cameras to directly acquire RGB-D images with depth information [47]. However, depth cameras, such as Kinect, cannot acquire usable data because of the varying light intensity in the field, necessitating radar and image fusion.

Jin et al. [46] used Faster Region Convolutional Neural Network to locate stalk positions in seedling maize populations. In that study, single plant segmentation of seedling maize population plants with plant height extraction was achieved via region growth algorithm. However, leaf crossing, shading, or overlapping can be problematic in the later stages of maize growth and development. Besides, it is difficult to segment single maize plants from the population point cloud. The accuracy of the segmentation algorithm directly determines the accuracy of the phenotype extraction results. In this study, a fast displacement algorithm was used to segment individual plants quickly and accurately. In combination with our related work [36,48], we were able to achieve the segmentation of maize leaves and stems. A point cloud segmentation pipeline from maize population to individual plants and to plant organs can be achieved. However, future studies should combine high-throughput data acquisition, data management, data processing, and phenotype extraction processes for high time-series plant phenotype analysis [5,13].

### Time-series images improve the accuracy of point cloud analysis

LiDAR has shown great potential in plant phenomics research [13]. The linear LiDAR can be calculated from time and velocity differentials based on inertial guidance, Global Positioning System, and other information to create a specific point cloud. However, the point clouds may not be sufficiently accurate since there may be differences in the coordinates of the data obtained at different growth stages. In this study, a fusion of orthorectified images and 3D point clouds effectively solved the time-series point cloud alignment problem. The simultaneously acquired orthorectified RGB images improved point cloud alignment accuracy.

The feature point finding limits the use of images to align point clouds. In this study, the point cloud data were aligned with RGB images and point clouds using DLT. Kang et al. [49] obtained desired alignment results based on a one-to-one correspondence of all pixel points in the image with points in the point cloud. However, the method consumed huge computational resources and was time-consuming. Therefore, abandoning the global pixel-by-pixel correspondence and using key feature points can reduce the computational resource consumption in the alignment process and improve the alignment efficiency of the LiDAR point cloud. Besides, critical issues in the top-view images of maize populations are relatively easier to find compared with point cloud alignment studies of unstructured data [24]. The outline of each maize plant and its centroids can be used as salient features for point cloud alignment. Moreover, previous work has conducted plant profile and centroid extraction [11].

### Field rail-based platform enables high-throughput and time-series phenotyping

High throughput is essential for the study of maize plants. Previous studies showed that linear LiDAR can provide high-quality point cloud data, allowing accurate phenotyping at multiple biological levels. Compared with terrestrial LiDAR, linear radar can improve data acquisition efficiency and enhance the throughput of 3D plant phenotyping studies. Many traits have been accurately extracted from radar data during data processing, such as crop height [50,51], canopy size [29], and volume [13]. The introduction of cutting-edge deep learning techniques has facilitated the HTP at the organ level, such as leaf inclination. This technological advance improves the accuracy of structural feature rhythm studies. Unmanned aerial vehicle (UAV) and unmanned ground vehicle (UGV) have also been used for HTP in some studies [22,52]. However, field rail-based phenotyping platforms are timely and acquire better quality data. Both UAV and UGV cannot simultaneously meet high timing and precision for data acquisition and analysis. The UAV cannot repeatedly fly without interruption due to their range. Moreover, UGV cannot obtain high-quality data because of the instability in maize fields, especially in the late stages of growth. However, the fixed coverage of phenotypic platforms limits the development of related research. For example, the platform has a limited monitoring range due to poor mobility. Therefore, a larger and more stable architecture is necessary.

Time-series analysis is also essential for studying plant phenotypes. Previous studies focused on structural traits at critical reproductive stages. Recent studies have shown that 3D structural data can be obtained at much shorter time intervals (high time series) to quantify plant growth dynamics. These studies have produced large datasets with very high storage and processing capacity requirements [13], putting greater demands on the processes and algorithms for point cloud processing. In this study, maize point cloud data (lasting 60 days) were tracked and analyzed to investigate the physiological mechanisms of maize growth and development. Therefore, this study provides insights into resolving temporal and topological changes during maize development. Phenotypic parameters of temporal dynamics can have broader application prospects in fields such as crop growth models or structure-function models.

### Limitations of the method

This method was oriented to the data acquired using the rail-based field phenotyping platform and, thus, highly depends on the data quality. Since the field weather conditions affect the raw data, especially the RGB images, we acquired the data twice in each day to capture satisfactory data as possible. In this study, we select high-quality data every several days and obtained good results. It is difficult to present growth dynamics every day due to the weather conditions.

Furthermore, the main phenotypic traits were derived from 3D point clouds. As for the acquisition by LiDAR, the point cloud of maize would be partially lost due to leaf crossing, shading, and overlapping during data acquisition. The point cloud loss would affect the final phenotypic measurement. High planting densities may affect the accuracy of the phenotype extraction. If the LiDAR could not capture the connection morphometrics among plant organs, the method is unable to segment the organs from individual plants. Therefore, the proposed method is applicable to maize grown at an appropriate density (the row spacing was 60 cm and plants were separated 30 cm of each row in the present study). It would also be interesting to design field trials of different densities to determine the limitations of the proposed method. We can even use the digital plant phenotyping platform [15] to simulate maize point clouds for different planting densities, which can be treated as a benchmark to verify the limitations of the proposed method.

In summary, the data quality of point cloud and RGB images was largely affected by planting density and weather conditions, respectively. Both fall under the category of suitable working conditions for the study of HTP platforms. This will be of great interest for plant phenomics.

In this study, the dynamics of phenotypic parameters in field maize populations were accurately estimated using the proposed methods. Alignment errors in time-series point cloud data were minimized by coupling field orthorectified images and point clouds. The proposed method integrates point cloud data acquisition, alignment, filtering, and segmentation algorithms. The method was robust under various conditions (different growth stages) and can be used in time-series dynamic phenotyping studies. The method can also be used to compare growth rates between cultivars or estimate botanical traits, which are features of interest to crop modelers and breeders. Therefore, this research can provide data supporting modern breeding.

## Data Availability

The data could be given upon reasonable request from the corresponding author.
